# Spatial Resolution Limit for Nanoindentation Mapping on Metallic Glasses

**DOI:** 10.3390/ma15186319

**Published:** 2022-09-12

**Authors:** Tao Liang, Qing Yu, Ziliang Yin, Songyi Chen, Ye Liu, Yanping Yang, Hongbo Lou, Baolong Shen, Zhidan Zeng, Qiaoshi Zeng

**Affiliations:** 1 Jiangsu Key Laboratory of Advanced Metallic Materials, School of Materials Science and Engineering, Southeast University, Nanjing 211189, China; 2 Center for High Pressure Science and Technology Advanced Research, Shanghai 201203, China

**Keywords:** metallic glass, heterogeneity, nanoindentation, spatial resolution limit

## Abstract

Spatial heterogeneity, as a crucial structural feature, has been intensively studied in metallic glasses (MGs) using various techniques, including two-dimensional nanoindentation mapping. However, the limiting spatial resolution of nanoindentation mapping on MGs remains unexplored. In this study, a comprehensive study on four representative MGs using nanoindentation mapping with a Berkovich indenter was carried out by considering the influence of a normalized indentation spacing *d*/*h* (indentation spacing/maximum indentation depth). It appeared to have no significant correlation with the measured hardness and elastic modulus when *d*/*h* > 10. The hardness and elastic modulus started to increase slightly (up to ~5%) when *d*/*h* < 10 and further started to decrease obviously when *d*/*h* < 5. The mechanism behind these phenomena was discussed based on a morphology analysis of residual indents using scanning electron microscopy and atomic force microscopy. It was found that the highest spatial resolution of ~200 nm could be achieved with *d*/*h* = 10 using a typical Berkovich indenter for nanoindentation mapping on MGs, which was roughly ten times the curvature radius of the Berkovich indenter tip (not an ideal triangular pyramid) used in this study. These results help to promote the heterogeneity studies of MGs using nanoindentation that are capable of covering a wide range of length scales with reliable and consistent results.

## 1. Introduction

Metallic glasses (MGs) have many superior physical and mechanical properties relative to conventional crystalline metallic materials due to their unique disordered atomic structure [1,2,3]. Very recently, spatial heterogeneity was realized as a critical structural feature in understanding the behavior and properties of MGs [4,5,6]. Various experimental and simulation methods were employed to investigate the heterogeneity in MGs at different length scales [7,8,9,10,11]. For instance, synchrotron X-ray diffraction (XRD) was used to explore the averaged structural heterogeneity at the atomic scale [8,12], and high-resolution transmission electron microscopy (HRTEM) was used to detect the nanometer scale density fluctuation in MGs [11,13,14]. In recent years, with the development of nanomechanical testing instruments, spatial heterogeneity was also characterized using the fluctuations of local mechanical properties via two-dimensional (2D) atomic force microscopy (AFM) mapping at the nanoscale [9,10,15] or nanoindentation mapping at the sub-micro- and microscale [16,17]. However, it is still challenging to directly compare the results derived from various techniques covering different spatial length scales to form a unified picture of the spatial heterogeneity of MGs. Therefore, a technique that can cover a wide range of length scales with reliable and consistent results is desirable.

Nanoindentation mapping is one of the most easily accessible and promising tools for acquiring the spatial heterogeneity information of MGs over a wide range of length scales [16,18,19,20,21]. Liu et al. reported a Zr-based MG with exceptional plasticity. They investigated the heterogeneity of the sample via nanoindentation mapping with a constant indentation depth of 500 nm and a spacing of 20 μm between the nearest indents [22]. Large fluctuations in the load at 500 nm were observed (from ~27 μN to ~34 μN), indicating hard and soft regions existing in the sample at the micrometer scale, which was proposed to account for the superplasticity of the sample [22]. A nanoindentation mapping study across a region of 0.8 mm × 1 mm of a Fe-based MG observed remarkable variations of the indentation depth (a change from 149.6 nm to 186.7 nm) and elastic modulus (Δ*E* up to 24.3%) in the sample, with a minimum distance of 100 μm between the adjacent indents [23]. An ultra-fast nanoindentation mapping technique was also used to characterize the structural heterogeneity in a Zr-based MG using a spherical indenter with a spacing of 100 nm between the adjacent indents [18]. A sudden or discontinuous change in the shear modulus, local viscosity, and relaxation time were observed in the mapping, which was attributed to the spatial distribution of solid- and liquid-like regions in the MG [18]. Nanoindentation tests are based on the elastic and plastic deformation of the materials beneath the tiny indenter tip; therefore, the spatial resolution of this method depends on the minimum size of the deformation-affected zone of a specific material. Although the heterogeneity can be observed at different length scales from ~100 nm to ~100 μm, the spatial resolution limit of nanoindentation mapping on MGs (considering their distinct deformation mechanism from conventional crystalline alloys) has not been clarified. Therefore, it is difficult to evaluate the experimentally observed spatial heterogeneity, especially in the sub-micron region [24].

In indentation experiments, a minimum spacing of three to five times the lateral dimension of the residual indent or a normalized spacing *d*/*h* (*d* is the distance between the centers of two nearest indents and *h* is the maximum indentation depth) above 20 has been followed as a criterion in various indentation tests for several decades [25,26,27]. This criterion is aimed to avoid interference from neighboring indents on the testing results because the plastic deformation introduced by indentation usually results in strain hardening in the material surrounding the indents. The requirement of a minimum spacing obviously limits the spatial resolution of nanoindentation mapping experiments. Interestingly, a recent study on the effect of spacing on several crystalline materials found that the measured hardness changed by only up to 5% when the normalized spacing *d*/*h* was reduced to 10 [26]. However, for MGs, the minimum *d*/*h* for reliable testing results in nanoindentation experiments has not been studied so far. The applicable normalized spacing parameter can be gainfully used to measure the local mechanical heterogeneity of MGs. In addition, this information is also essential for measuring the properties of small volume samples of, e.g., MG films [28], as well as detecting the sample size effect [29,30] or indentation size effect [31].

In this study, we investigated the effect of indent spacing on the measured elastic modulus and hardness in nanoindentation mapping experiments with a typical Berkovich indenter on four representative MG samples. The behavior of these MG samples was compared with that of prototype crystalline metals such as Cu and Ni. The morphology of the residual indents was characterized using scanning electron microscopy (SEM) and AFM to understand the effect of the plastic-deformation-induced pile-up on the nanoindentation results. A critical *d*/*h* value and spatial resolution limit for reliable nanoindentation mapping were obtained and successfully used to investigate the heterogeneity of the Fe_78_Si_9_B_13_ MG as a demonstration.

## 2. Materials and Methods

Systematic nanoindentation tests were conducted on four prototype MGs with different mechanical properties at room temperature: Zr_50_Cu_50_, Fe_78_Si_9_B_13_, Au_49_Ag_5.5_Pd_2.3_Cu_26.9_Si_16.3_, and Ce_60_Al_15_Cu_10_Ni_15_. Other standard materials, such as fused silica (SiO_2_) and polycrystalline nickel, were also used for comparison. MG ribbons ranging from 25 to 63 μm in thickness were prepared via melt-spinning on a rotating copper wheel under a Ti-gettered high-purity argon atmosphere from master alloys. Smooth free surfaces were obtained during the ribbon sample synthesis. The glass characteristics of the MGs were verified using differential scanning calorimetry (DSC, Perkin-Elmer 8500, Waltham, MA, USA) with a constant heating rate of 20 K/min and synchrotron XRD with an X-ray wavelength of ~0.6199 Å at beamline 15U1, Shanghai Synchrotron Radiation Facility (SSRF), Shanghai, China. The fused silica and polycrystalline nickel were standard commercial products and were mechanically polished to a mirror finish.

Nanoindentation experiments were conducted at room temperature using an instrumented nanoindentation system (KLA G200, Milpitas, CA, USA) with a Berkovich indenter calibrated on fused silica. A standard XP mode was applied with an indentation displacement resolution of 0.01 nm, a sample stage positioning accuracy of 1 μm, and a load resolution of 50 nN. A dynamic contact module (DCM) was applied with an indentation displacement resolution of 0.2 pm, a sample stage positioning accuracy of 20 nm, and a load resolution of 3 nN. Arrays of 6 × 6 or 10 × 10 indents were used in the indentation mapping by setting different maximum loads *P*_max_ or different distances *d* between adjacent indents. The maximum load *P*_max_ is the target load in each indenting process. For a given sample, the same *P*_max_ corresponded to an almost identical maximum penetrating depth *h*_max_. The thermal drift was kept below 0.05 nm/s and was corrected using measurements at 10% of the full load during unloading. In order to determine the minimum indentation depth of the Berkovich indenter required for reliable measurements, continuous stiffness measurement (CSM) was also used to acquire the elastic modulus as a function of the indentation depth. In total, data from more than 3000 individual nanoindentations were collected for further statistical analysis in this work. The morphology of the residual indent arrays was characterized using SEM (VERSA 3D, FEI, Hillsboro, OR, USA) and AFM (Asylum Research MFP-3D, Oxford Instruments, High Wycombe, Bucks, UK).

## 3. Results and Discussion

### 3.1. Effect of Spacing on the Nanoindentation Mapping Results

**Figure 1:**
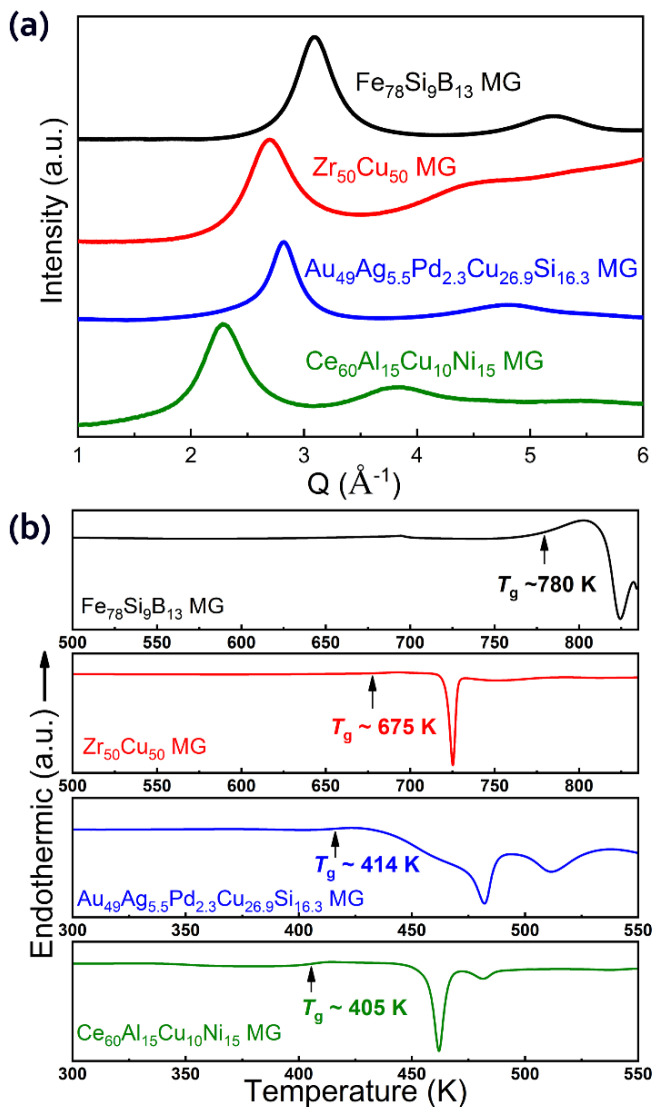
Characterization of the Fe_78_Si_9_B_13_, Zr_50_Cu_50_, Au_49_Ag_5.5_Pd_2.3_Cu_26.9_Si_16.3_, and Ce_60_Al_15_Cu_10_Ni_15_ (nominal compositions) metallic glass samples. (**a**) Synchrotron X-ray diffraction patterns of the four metallic glasses. (**b**) Differential scanning calorimetry traces of the four metallic glasses.

Figure 1 shows the synchrotron XRD patterns and DSC traces of the as-prepared MG samples. The diffuse peaks in the XRD patterns and endothermic glass transition signals in the DSC traces confirmed the fully amorphous features of all four MGs. These four samples were chosen as representative MGs with diverse mechanical properties at room temperature, e.g., yield strength, brittleness/ductility, bulk modulus, and hardness [32]. For MGs, a lower *T*_g_ usually corresponds with a lower hardness and elastic modulus [33]. The mechanical properties and the glass transition temperatures (*T*_g_) of these MGs are summarized in Table 1, in which experimental results from both the literature and the current study are included [22,34,35].

**Figure 2:**
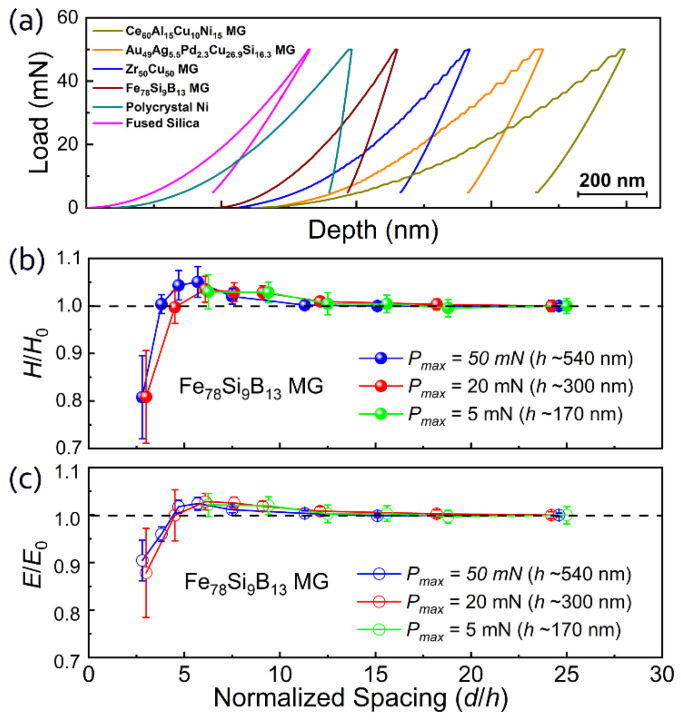
Nanoindentation tests. (**a**) Representative load–depth curves of nanoindentation tests on the Fe_78_Si_9_B_13_, Zr_50_Cu_50_, Au_49_Ag_5.5_Pd_2.3_Cu_26.9_Si_16.3_, and Ce_60_Al_15_Cu_10_Ni_15_ metallic glass samples; fused silica; and polycrystal nickel. The maximum load was 50 mN, and the loading rate was 1 mN/s. The curves are offset horizontally for clarity. (**b**) The normalized mean hardness (*H*/*H*_0_) and (**c**) normalized mean modulus (*E*/*E*_0_) of the Fe_78_Si_9_B_13_ metallic glass as a function of the normalized spacing (*d*/*h*) obtained using a 6 × 6 array nanoindentation mapping. *H*_0_ and *E*_0_ are the “normal” values obtained with a large normalized spacing (*d*/*h* ≈ 25).

Figure 2a shows the representative load–penetration depth (*h*) curves of the four MGs, polycrystal nickel, and fused silica with an identical experimental setup (*P*_max_ = 50 mN, and the loading rate was 1 mN/s). The loading curves of the fused silica and polycrystalline nickel were very smooth and continuous. In contrast, the four MGs showed typical “pop-in” events in the loading curves. The pop-ins in the loading curves correspond to the serrated flows induced by the activation of individual shear bands during indentation [36]. The deformation of MGs at temperatures far below *T*_g_ always occurs heterogeneously through the concentration of inelastic strains in localized shear bands [37,38], which is distinct from typical crystalline metals. Different “pop-in” sizes among the four MGs with an identical loading rate also reflected the differences in their mechanical properties [39].

Figure 2b,c show the variations in normalized mean hardness (*H*/*H*_0_) and normalized mean elastic modulus (*E*/*E*_0_) derived from the nanoindentation mapping on the Fe_78_Si_9_B_13_ MG with different *d*/*h* values. H and E are the mean hardness and elastic modulus values at different *d*/*h* values, respectively, while *H*_0_ and *E*_0_ are the respective “normal” values obtained with large normalized spacing (*d*/*h* ≈ 25). The elastic modulus and hardness remained almost constant for *d*/*h* ≥ 10. When *d*/*h* was below ~10, both the elastic modulus and hardness started to increase slightly with decreasing *d*/*h*. However, when *d*/*h* further decreased below ~5, the elastic modulus and hardness decreased sharply. In addition, for the experiments with three different load (*P*_max_)/indentation depths, the effect of spacing (*d*/*h*) on the measured elastic modulus and hardness was highly consistent, indicating that the impact of *P*_max_ can be discounted.

**Figure 3:**
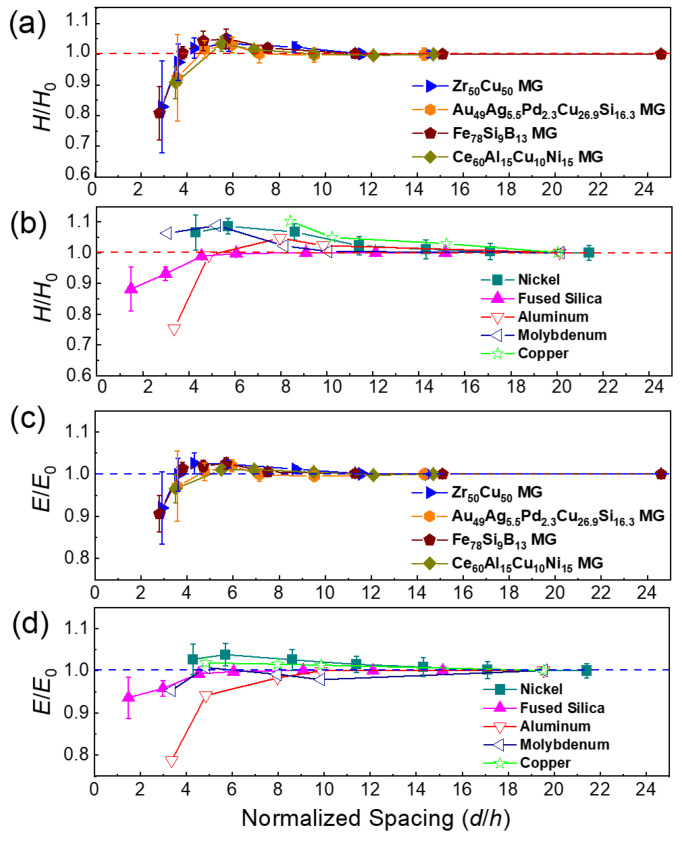
Mechanical properties determined using 2D nanoindentation mapping as a function of normalized spacing (*d*/*h*) between adjacent indents. The normalized hardness (*H*/*H*_0_) as a function of *d*/*h* for four metallic glass samples (**a**), as well as crystalline metals and fused silica (**b**). The normalized elastic modulus (*E*/*E*_0_) as a function of *d*/*h* for four MG samples (**c**) and crystalline metals (**d**). Each data point in the current study (solid symbols) was obtained by averaging the results of a 6 × 6 array in nanoindentation mapping with a specified *d*/*h*, while *H*_0_ and *E*_0_ were the “normal” values obtained with the maximum normalized spacing. The data for aluminum, molybdenum, and copper (open symbols) are from [26].

We further investigated the effect of spacing on the nanoindentation results in the other three MG samples and other standard materials, including polycrystalline Ni and fused silica (Figure 3). As shown in Figure 3a,c, the results of the other three MG samples were similar to those observed in the Fe_78_Si_9_B_13_ MG. The critical values for the deviation in different MGs were summarized in Table 2. Overall, the change in elastic modulus was smaller than that of the hardness. Compared with the MG samples, the hardness and elastic modulus variation in crystalline metals were more noticeable when the spacing between adjacent indents decreased (see Figure 3b,d). For instance, the measured hardnesses of Cu and Ni increased by 5.0% and 2.3%, respectively, when *d*/*h* was ~10.0; increased by 10.3% for Cu when *d*/*h* = 8.4; and increased by 8.7% for Ni when *d*/*h* = 5.7 [26]. While those of the MG samples remained almost unchanged (the average increase of 0.2% was much smaller than the standard deviation) when *d*/*h* was ~10.0 and increased by 3.0–5.1% when *d*/*h* was ~5.7. The MGs seemed less sensitive than crystalline metals to the decrease in *d*/*h*. Therefore, a smaller *d*/*h* can be used for nanoindentation mapping experiments on MGs compared with crystalline metals, which could be attributed to a relatively more localized deformation-affected zone in the nanoindentation of MGs [37].

### 3.2. Morphological Analysis of the Indents

**Figure 4:**
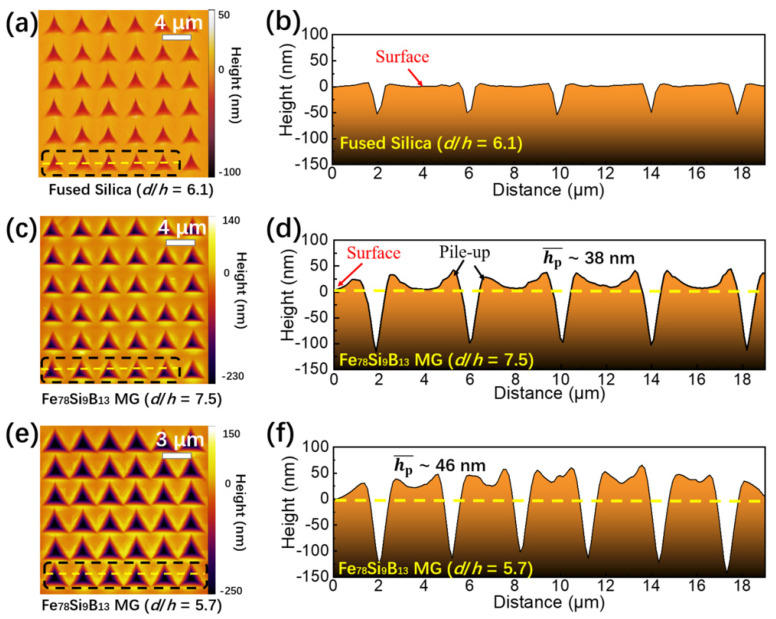
Morphology of the residual indentations obtained via atomic force microscopy analysis. (**a**) Height distribution of a 6 × 6 array on the fused silica (*d*/*h* = 6.1) using atomic force microscopy scanning and (**b**) the corresponding profile of the indentations in the array along a line indicated in (**a**) within the black dotted box. Similar analyses of the Fe_78_Si_9_B_13_ metallic glass (*d*/*h* = 7.5 and 5.7) are presented in (**c**–**f**). The horizontal dashed lines in (**b**,**d**,**f**) represent the positions of the original sample surface before indentation. The pile-up height was defined as the maximum height above the original sample surface.

Pile-up around each indent has been widely observed in nanoindentation experiments of MGs due to their plastic deformation. This causes an underestimation of the real contact area and leads to higher hardness and elastic modulus in the nanoindentation measurements [40]. The hardness obtained by nanoindentation is the calculated average contact pressure (the load divided by the contact area) [27]. Since the load values can be accurately obtained, the accuracy of the contact area determined by the instrument plays an important role in the hardness calculation. With decreasing spacing between the indents, the plastic deformation zone around each indent will become closer and eventually overlap, enhancing the pile-up effect. Therefore, we used AFM to obtain profiles of the residual indents to obtain quantitative information on the pile-up in the Fe_78_Si_9_B_13_ MG with relatively small *d*/*h* values, for which the deviation in the hardness and elastic modulus was most noticeable. As shown in Figure 4b, the surface between the indents of the fused silica remained mostly flat after indentation experiments with *d*/*h* = 6.1 without any pile-ups due to pressure-induced permanent densification [41]. In contrast, the Fe_78_Si_9_B_13_ MG showed significant pile-up after indentation experiments, i.e., the average height of the pile-ups (*h*_p_) was ~38 nm when *d*/*h* = 7.5 and ~46 nm when *d*/*h* = 5.7 (see Figure 4d). For experiments with indents far apart from each other (*d*/*h* = 24.6), *h*_p_ was much smaller (~28 nm). Therefore, the observation that *h*_p_ increased when the indents were closer seemed to be closely associated with the overlap of pile-up regions. As mentioned previously, pile-up would lead to higher hardness in nanoindentation experiments; hence the higher pile-up may explain the increased hardness when the spacing between indents was small, e.g., *d*/*h* < 10. Furthermore, the effect of pile-up can be estimated quantitatively based on the fact that the contact area between the indenter and the sample was proportional to the square of the actual indentation depth, which was the sum of the nominal indentation depth (as shown in the load–depth curve) and the height of the pile-up. Compared with the case in which the indents were far apart (*d*/*h* = 24.6), the contact area increased by 4% and 8% for *d*/*h* = 7.5 and *d*/*h* = 5.7, respectively. These values were consistent with the corresponding hardness increase of 2.0% and 5.1% measured for *d*/*h* = 7.5 and *d*/*h* = 5.7, respectively. Therefore, the increased hardness of MGs could be mainly attributed to the effect of plastic deformation-induced pile-up. Other effects, such as plastic-deformation-induced softening in the deformation-affected zone underneath the indents in MGs, may have played a minor role, which accounted for the slightly decreased hardness compared with that estimated from contact area increases caused by pile-ups.

**Figure 5:**
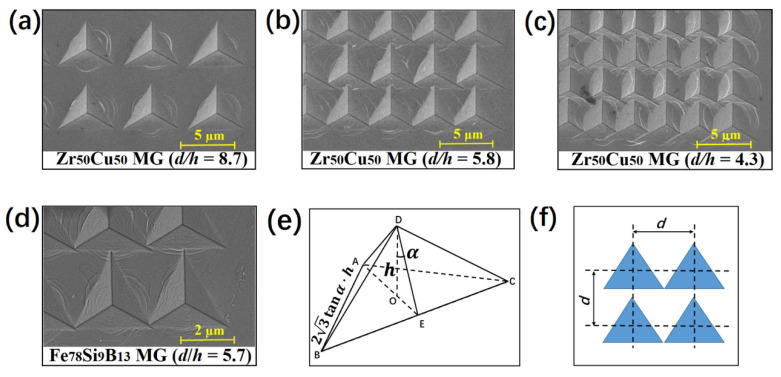
Geometric analysis of the indent array in the nanoindentation mapping. (**a**–**c**) Scanning electron microscopy images of the indent array on the Zr_50_Cu_50_ metallic glass with *d*/*h* = 8.7, 5.8, and 4.3, respectively. (**d**) Scanning electron microscopy image of the indents on Fe_78_Si_9_B_13_ metallic glass after nanoindentation mapping with *d*/*h* = 5.7. (**e**) Schematic of the Berkovich indenter tip. OD is the height *h* of the tri-pyramid ABCD, which indicates the indenting displacement beneath the sample surface, and ΔABC is an equilateral triangle. ∠ODE is the processing angle *α* of the standard Berkovich tip, which was ~65.3°. (**f**) Schematic of the critical spacing distance when the indentations started overlapping.

When the normalized spacing between the indents shrunk further (*d*/*h* < 5), all materials’ measured hardnesses and elastic moduli decreased. Hardness reduction of up to 20% was observed when *d*/*h* < 3. This dramatic drop could be attributed to the severe overlap of the indents (Figure 5a–c), which essentially reduced the actual contact area in the indentation and also resulted in deviation during the detection of the zero point of the indentation depth. According to the geometry of the Berkovich indenter, the critical normalized spacing *d*/*h* below which the indents would overlap was calculated to be 7.5 when only considering plastic deformation in the indentation (see Figure 5e,f). Due to the contribution of elastic deformation, the actual critical value depended on the displacement recovery ratio *h*_f_/*h*_m_ (*h*_m_ is the maximum penetration depth and *h*_f_ is the residual indentation depth) [40]. According to the calculation based on the load–depth curves of some representative MGs, this ratio is usually in the range of 0.7~0.8 for MGs [42,43]; hence the critical *d*/*h* should be 5.3~6.0. This was consistent with our experimental observation in Figure 4 and Figure 5.

### 3.3. Mechanism behind the Spacing Limits

As mentioned previously, our results suggested that a smaller *d*/*h* can be used for the nanoindentation mapping on MGs compared with crystalline metals. The increased hardness in experiments with 5 < *d*/*h* < 10 could mainly be attributed to the effect of enhanced pile-up due to approaching adjacent indents. The difference probably resulted from the different plasticity mechanisms in crystalline and amorphous alloys. For crystalline metals and alloys, plastic deformation mainly takes place through the gliding of dislocations, and substantial strain or work hardening is widely observed due to the strong interaction and tangling between dislocations, which slows further plastic deformation. In contrast, for MGs, shear banding is widely accepted as the main mechanism of room temperature plasticity [44,45]. Moreover, MGs usually show strain softening rather than hardening due to the soft region introduced during the shear deformation [45,46,47,48]. According to our results, we did not observe obvious evidence for strain hardening in the four MG samples since the pile-up can reasonably account for the increased hardness in experiments with 5 < *d*/*h* < 10. For example, when *d*/*h* was reduced to 5.7, the measured hardness of the Fe_78_Si_9_B_13_ MG was expected to rise by ~8%. However, if strain hardening was present and the pile-up effect is considered, the total hardness increase should have been much more significant than 8%. Yet, in experiments, the hardness of Fe_78_Si_9_B_13_ MG only rose by 5.1%. Therefore, hardness slightly lower than the expected value might have been attributed to the slight strain softening effect in MGs.

### 3.4. Minimum Spacing for Nanoindentation Mapping

**Figure 6:**
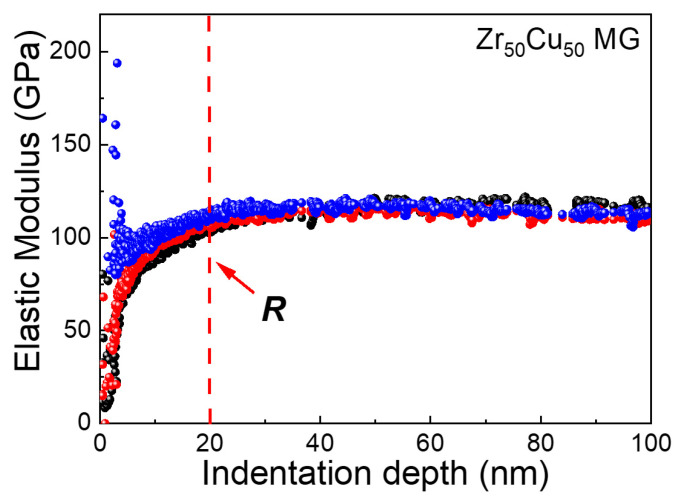
Elastic moduli of the Zr_50_Cu_50_ metallic glass sample as a function of indentation depth during nanoindentation using the continuous stiffness measurement technique. The sample data were from three independent nanoindentation tests showing good reproducibility. The vertical dashed line marks the radius of the Berkovich indenter tip curvature value *R*.

Since the actual spacing *d*/*h* between indents depends both on the spacing *d* and the maximum indentation depth *h*, we also need knowledge of the minimum penetration depth in order to obtain the highest spatial resolution *d* in the 2D mapping of nanoindentation. In practice, the tip of a Berkovich indenter cannot be an ideal triangular pyramid; instead, it is approximately part of a sphere [49]. The radius of the Berkovich indenter tip curvature *R* was ~20 nm in our experiments. This means that the shape of the indenter was spherical rather than a pyramid when the indentation depth was below 20 nm, which was consistent with our observation that stable values of elastic moduli only appear when the indentation depth was increased above ~20 nm (Figure 6). Even when the *R* value is unknown for a given indenter due to tip blunting, it can be readily estimated using the critical indentation depth for stable elastic moduli, as shown in Figure 6. Therefore, for the Berkovich indenter we used in this work, a minimum indentation depth of 20 nm was required for a reliable nanoindentation test on the MG samples. Considering both the minimum indentation depth required and minimum normalized spacing (*d*/*h* = 10), a spatial resolution of 200 nm can be achieved in the nanoindentation 2D mapping by using a Berkovich indenter *R* of ~20 nm.

**Figure 7:**
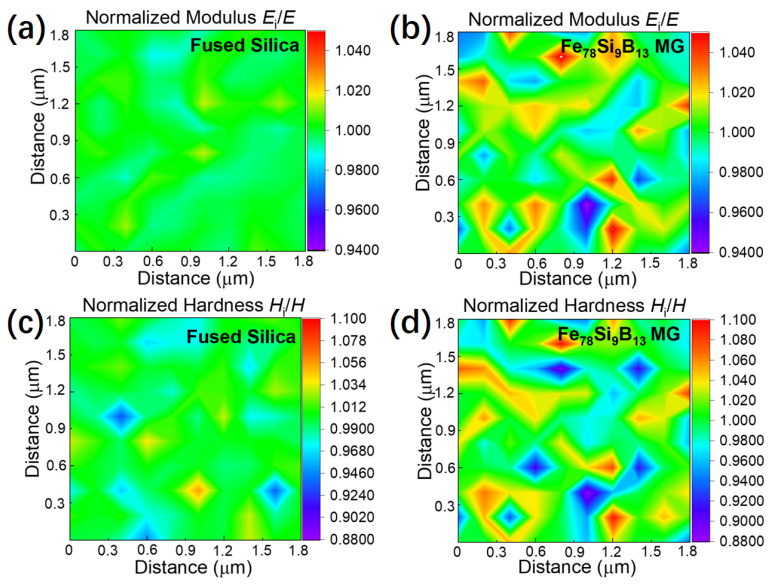
The 2D distribution of mechanical properties obtained via nanoindentation mapping. Normalized elastic modulus *E*_i_/*E* distribution maps of (**a**) fused silica and (**b**) Fe_78_Si_9_B_13_ MG. Normalized hardness *H*_i_/*H* distribution of (**c**) fused silica and (**d**) Fe_78_Si_9_B_13_ metallic glass. All the data were normalized using their average values (*E* or *H*) in the 2D mapping. A 10 × 10 array indentation mapping was employed with a spacing (*d*) of 200 nm and a maximum depth (*h*) of 20 nm across a region of 1.8 × 1.8 μm^2^. The variations of both elastic modulus and hardness are shown using the color scales.

As a demonstration, we used these parameters to investigate the heterogeneity in the Fe_78_Si_9_B_13_ MG via nanoindentation 2D mapping with the highest spatial resolution available. A standard material, namely, fused silica, is also presented with an identical experimental parameter for comparison. The hardness and elastic moduli were normalized using their average values to better compare the relative fluctuations in properties. As shown in Figure 7a,c, the normalized elastic modulus (*E*_i_/*E*) and hardness (*H*_i_/*H*) were relatively uniform across the tested area for the fused silica, e.g., the overall standard deviation was ~0.50% for the elastic moduli. In contrast, the fluctuation was more noticeable for the Fe_78_Si_9_B_13_ MG (see Figure 7b,d), especially in the hardness distribution, revealing obvious heterogeneity of the Fe_78_Si_9_B_13_ MG with characteristic sizes of approximately 200–300 nm. Due to the apparent heterogeneity in the Fe_78_Si_9_B_13_ sample, the standard deviations of mapping data of the elastic modulus and hardness were ~2.31% and ~4.68%, respectively, which were much higher than the ~0.50% and ~1.56% in the fused silica control sample.

The spatial heterogeneity in the Fe-based MGs was investigated in previous studies. A nanoindentation mapping study across a 1.0 × 0.8 mm^2^ area of a Fe_50_Ni_30_P_13_C_7_ bulk MG with a spacing of ~200 μm reported a hardness fluctuation of ~8.2% (the average hardness was 6.84 ± 0.56 GPa) [23]. A maximum indentation depth of around 200 nm was used in that study, suggesting that the tested area size was larger than 1 μm for each indent. Therefore, the results implied heterogeneity in the Fe_50_Ni_30_P_13_C_7_ sample at the micron scale. On the other hand, amplitude-modulation dynamic AFM studies on Fe_80_Si_9_B_11_ and Fe_80−x_M_x_Si_9_B_11_ (M = Co and Ni, x = 0, 2, 4) MGs suggested liquid-like regions with an average size of ~10 nm [50,51]. Our results in this work revealed heterogeneity with a characteristic domain size of ~200–300 nm by pushing the spatial resolution of regular nanoindentation tests down to 200 nm, providing the missing information about heterogeneity at the intermediate length scale and implying that the distribution of those “liquid-like regions” was also not uniform at the hundreds of nanometers scale in the Fe-based MG.

## 4. Conclusions

In summary, a comprehensive study was carried out to clarify the effect of the normalized spacing *d*/*h* between adjacent indents on the results of nanoindentation mapping experiments on various MGs. Our results suggested that compared with conventional crystalline metals, smaller normalized spacing can be used in nanoindentation 2D mapping experiments on MGs due to their relatively localized affecting zone of plastic deformation. Specific conclusions are listed as follows for the nanoindentation mapping on MGs:

(1) The measured hardness and elastic modulus showed no significant dependence of *d*/*h* with *d*/*h* > 10 and were independent of the load when *h* (maximum indentation depth) > *R* (the radius of the Berkovich indenter tip curvature).

(2) The hardness and elastic modulus increased slightly (up to ~5%) when 5 *< d*/*h* < 10, which could mainly be attributed to the effect of enhanced pile-ups. The hardness and elastic modulus obviously decreased when *d*/*h* further decreased below 5 due to the severe overlap between the adjacent indents.

(3) By optimizing the experimental parameters, a spatial resolution of ~200 nm can be achieved when detecting the fluctuations of the local mechanical properties of MGs with *R* ≈ 20 nm. A demonstration on a Fe_78_Si_9_B_13_ sample revealed heterogeneity at a scale of ~200–300 nm. In principle, a higher spatial resolution (~10*R*) can be achieved if a smaller radius of curvature (*R*) of the Berkovich indenter tip is available.

Our findings help to promote studying heterogeneity in MGs at the mesoscale using nanoindentation for reliable and consistent results.

## Figures and Tables

**Table 1:** Mechanical and physical parameters (*σ*_y,_ yield stress; *ν*, Poisson’s ratio; *E*, elastic modulus; *H*, hardness; *T*_g_, glass transition temperature) of the four metallic glasses.

Sample	*σ*_y_ (MPa)	*ν*	*E* (GPa)	*H* (GPa)	*T*_g_ (K)
Fe_78_Si_9_B_13_ (this work)	-	-	183 ± 2	8.8 ± 0.1	~710
Zr_50_Cu_50_ [34]	1272	0.365	92 ± 2	5.9 ± 0.2	~670
Ce_60_Al_15_Cu_10_Ni_15_ [35]	1200	0.328	78 ± 2	4.6 ± 0.1	~400
Au_49_Ag_5.5_Pd_2.3_Cu_26.9_Si_16.3_ [22]	705	0.406	45 ± 1	2.4 ± 0.1	~410

**Table 2:** The critical normalized spacing and corresponding changes of the hardness and elastic modulus of the four tested metallic glasses and fused silica relative to the “normal” values obtained with a large normalized spacing (*d*/*h* ≈ 25).

Sample	Normalized Spacing, *d*/*h*	Change of Hardness (%)	Standard Deviation	Change of Modulus (%)	Standard Deviation
Fe_78_Si_9_B_13_ MG	5.7	5.1	3.2	2.6	1.3
Zr_50_Cu_50_ MG	5.8	3.5	2.6	2.5	0.9
Ce_60_Al_15_Cu_10_Ni_15_ MG	5.5	3.4	2.4	1.1	0.9
Au_49_Ag_5.5_Pd_2.3_Cu_26.9_Si_16.3_ MG	6.0	3.0	2.5	2.0	1.5
Fused silica	6.1	−0.2	0.8	−0.2	0.4

## Data Availability

The data that support the findings of this study are available from the corresponding author upon reasonable request.
